# Intractable Singultus: Causes, diagnosis and treatment. A case report

**DOI:** 10.1192/j.eurpsy.2024.1029

**Published:** 2024-08-27

**Authors:** C. Díaz Mayoral, E. Arroyo Sánchez, A. Martínez Pillado, P. Setién Preciados

**Affiliations:** ^1^Psiquiatría, Hospital Universitario Príncipe de Asturias; ^2^Psiquiatría, Hospital Universitario Rey Juan Carlos, Madrid, Spain

## Abstract

**Introduction:**

Hiccups are involuntary, spasmodic contractions of the diaphragm and intercostal muscles that cause inspiration and are interrupted by closure of the glottis. Most sources define the term “persistent hiccups” as lasting more than 2 days and “intractable” as lasting more than 1 month. Both are most likely associated with a pathologic process. “Intractable hiccups” should lead to investigation of organic pathology. If it does not improve, it can interfere with the patient’s ability to eat, socialize and sleep, leading to a significant worsening of quality of life. “Intractable hiccups” are more frequent in men (91%), over 50 years of age. Women suffer from psychogenic hiccups more frequently than men. Anxiety or stress can trigger hiccups. Multiple neurotransmitters are involved.

**Objectives:**

We present a theoretical review on the topic.

**Methods:**

A bibliographic review on the topic.

**Results:**

In recent years, new trials and case series have been published, and regulatory agencies have issued new recommendations on the use of pharmacologic agents for this indication. The literature has described the efficacy of several pharmacologic agents in the empiric treatment of persistent and intractable hiccups. Most of these target dopaminergic and GABAergic receptors.

Based on limited efficacy and safety data, Baclofen and Gabapentin can be considered as first-line treatment for intractable and persistent hiccups, as they suggest efficacy and are less likely to cause long-term side effects than standard neuroleptic agents. Dopamine blocking agents such as Metoclopramide, Chlorpromazine, and Haloperidol, could be used as second line. In one study, withdrawal of Benzodiazepines or addition of Pregabalin was found to help reduce hiccups.

The patient we consulted came for persistent hiccups or singultus of 2 years of evolution. Organic pathology was ruled out. She related the onset of the symptoms to different stressors that had caused her anxiety. We administered Escitalopram and Gabapentin and indicated withdrawal of Bromazepam, which she started taking months ago. In follow-up appointments she reported a decrease in the intensity and frequency of the symptoms, with a notable improvement in her quality of life.

**Conclusions:**

Considering all available evidence, a treatment algorithm with Baclofen is recommended as first-line therapy for persistent and intractable hiccups. Gabapentin may also be safe and effective in the long-term treatment of this condition, especially for patients with CNS disease. Metoclopramide is no longer recommended for long-term treatment of hiccups. Clinical experience also supports the use of Chlorpromazine and other neuroleptics for acute, but not long-term, treatment. Going forward, large multicenter studies will be needed to create an adequate evidence base for the treatment of persistent and intractable hiccups. Until then, guidelines will continue to be based on unreliable data and clinical experience.

**Disclosure of Interest:**

None Declared

